# Brief Psychological Intervention Through Mobile App and Conference Calls for the Prevention of Depression in Non-Professional Caregivers: A Pilot Study

**DOI:** 10.3390/ijerph17124578

**Published:** 2020-06-25

**Authors:** Patricia Otero, Isabel Hita, Ángela J. Torres, Fernando L. Vázquez

**Affiliations:** 1 Department of Psychology, University of A Coruña, 15071 A Coruña, Spain; 2 Department of Clinical Psychology and Psychobiology, University of Santiago de Compostela, 15782 Santiago de Compostela, Spain; isabel.hita@usc.es (I.H.); fernandolino.vazquez@usc.es (F.L.V.); 3 Department of Psychiatry, Radiology and Public Health, University of Santiago de Compostela, 15782 Santiago de Compostela, Spain; angelajuana.torres@usc.es

**Keywords:** depression, nonprofessional caregiver, prevention, cognitive, behavioral, telephone, app

## Abstract

Despite its potential, no intervention aimed at non-professional caregivers administered through a smartphone app has been proven to prevent depression. The objective of this pilot study was to evaluate the efficacy and feasibility of an indicated depression-prevention intervention for non-professional caregivers administered through an app with the addition of conference-call contact. The intervention was administered to 31 caregivers (Mean age = 54.0 years, 93.5% women). An independent evaluation determined the incidence of depression, depressive symptoms, risk of developing depression, and the variables in the theoretical model (positive environmental reinforcement, negative automatic thoughts) at the pre-intervention and post-intervention, as well as the one- and three-month follow-ups. The incidence of depression at 3 months of follow-up was 6.5%. There was a significant reduction in depressive symptoms (*p* < 0.001) and in the risk of developing depression (*p* < 0.001) at the post-intervention and at the one- and three-month follow-ups. The model’s variables improved significantly after the intervention and were associated with post-intervention depressive symptoms. The intervention was more effective in caregivers who had a lower level of depressive symptoms at the pre-intervention. Adherence and satisfaction with the intervention were high. The results encourage future research using a randomized controlled clinical trial.

## 1. Introduction

Caregivers for dependent family members perform a job that requires them to meet many demands and cope with difficult situations, oftentimes over a long period of time [1], such as looking for a diagnosis, learning to care for their family member, financial matters [2], decreased environmental reinforcement [3], insomnia or low-quality sleep [4,5], or a decrease in capabilities of the family member [6]. Therefore, caregivers’ quality of life is affected [7,8,9], and these risk factors can increase their vulnerability to mental health disorders, especially depression [10]. In fact, a systematic review and meta-analysis on ten studies with a total of 790 caregivers [11] found a relative risk ranging from 2.80 to 38.68 for suffering from a depressive disorder in caregivers compared to non-caregivers. Specifically, previous research has found that 8.9% of caregivers meet the criteria for an episode of major depression [12], which is nearly fivefold higher than the 1.8% prevalence of major depression in the general population [13]. These figures are quite important because major depression is highly disabling and constitutes the third-leading cause of years lived with disabilities [14]. In addition, it is recurrent in 27% to 42% of people who experienced one major depressive episode and symptoms are chronic in 12% to 16% of those who have developed clinical depression [15,16,17]. Likewise, it entails enormous socioeconomic costs, resulting in billions of dollars annually [18]. Furthermore, in the case of caregivers, it can interfere with the proper performance of their tasks in caring for the dependent person [19].

Therefore, given the prevalence and serious repercussions of depression, its prevention among the caregiver population is critical. Among preventive strategies, indicated prevention interventions are particularly interesting. These are interventions aimed at individuals with subclinical symptoms who do not meet the diagnostic criteria for a major depressive episode [20]. The goal is to reduce the progression of these symptoms and to prevent their worsening into major depression [21].

There are only three previous studies on the indicated prevention of depression in non-professional caregivers providing care for dependent family members. The first evaluated the efficacy of a five-session problem-solving intervention using a group face-to-face format (about five participants per group) compared to a usual-care control group. It found a lower incidence of depression (4.5% vs. 13.1%) and fewer depressive symptoms (*d* = 1.54) after the intervention [22] and at the 12-month [23] and eight-year follow-up [24]. The second evaluated the efficacy of a five-session cognitive-behavioral face-to-face group intervention (about five participants per group) compared to a usual-care control group. It found a lower incidence of depression (1.1% vs. 12.2%) and fewer depressive symptoms (*d* = 1.05) after the intervention [25] and at the 12-month follow-up [26]. The therapeutic changes found were clinically significant [27]. However, these interventions were conducted using a traditional face-to-face format, which could compromise their accessibility. Caregivers often have barriers that make it difficult for them to attend face-to-face interventions, including lack of time, a lack of a substitute caregiver in their absence, a shortage of mental health services, travel and cost concerns, and fear of stigmatization.

The use of communication technologies available to practically all caregivers, such as the telephone, reduces these barriers. The telephone offers travel savings, anonymity, lower cost, and accessibility to the most remote communities [28], in addition to solving specific barriers in the caregiver population, such as lack of time and difficulties in finding a substitute caregiver. Therefore, in the third study of indicated depression prevention aimed at caregivers, Vázquez et al. (2020) [29], took a cognitive-behavioral depression-indicated prevention intervention that had previously been tested in a face-to-face format and adapted it into a five-session conference call format. The study also examined the efficacy of its components using a dismantling strategy. They found that the incidence of depression was lower in the group that received either the complete conference call intervention or the behavioral activation component only, compared to the control group (1.5% and 1.4% vs. 8.8%), and depressive symptoms were significantly lower in both intervention groups compared to the control group (*d* = 1.16 and *d* = 1.29), with no differences between them.

Although these results were very promising, the prevention intervention for depression could be optimized by utilizing a smartphone app. Currently, it is estimated that more than five billion people worldwide have mobile devices and more than half of these connections are smartphones. Furthermore, 80% of adults in Spain own a smartphone or use apps [30]. The smartphone app allows the caregiver greater mobility and accessibility by allowing access wherever and whenever they want 24 h a day. It increases the flexibility of program participation and can be adapted to caregivers’ personal routines without wait times, having to make appointments, or attend sessions at a fixed time. Users can also review the materials as many times as they want and interact and receive feedback immediately [31,32].

However, given its novelty, there are only two previous studies evaluating apps for the treatment of depression [33,34], and neither of them are aimed at preventing depression or are aimed at caregivers of dependent family members. In particular, Arean et al. (2016) [33] found no significant difference at the post-intervention time point and the one-month follow-up between a cognitive training intervention, another problem-based intervention, and an attention control group in which participants received therapeutically inactive information on health. At the three-month follow-up, participants in both interventions showed higher remission rates compared to controls (50% and 49% vs. 32%), but only those with moderate depressive symptoms who participated in the problem-solving group showed fewer depressive symptoms than the control group (*d* = 0.76). In addition, Ly et al. (2014) [34] found no post-intervention differences between a program based on behavioral activation and another based on mindfulness. At the six-month follow-up, the behavioral activation intervention was more effective for patients with greater initial severity of depression (*d* = 0.47), while the mindfulness intervention was more effective for patients with lower severity (*d* = 0.98).

However, lack of adherence is one of the main limitations of interventions that do not use a face-to-face format. Dropout rates for interventions using apps were high, reaching 31.6%, and the level of compliance with tasks between sessions is unknown [33]. These problems are critical, because when evaluating the efficacy of interventions, high dropout rates can lead to bias and limit the generalizability of the results [35]. Furthermore, task completion is a significant predictor of therapy outcomes in depression prevention interventions among the caregiver population [36]. One way to reduce drop-out rates in interventions that are not conducted in an in-person format would be to establish telephone contact [31]. In addition, using the positive and corrective feedback technique, which consists of reinforcing the correct performance of a behavior and providing instructions to change behaviors that have been conducted incorrectly [37], could increase the effectiveness of interventions and strengthen caregivers’ commitment to completing the homework tasks.

The objective of this study was to evaluate the efficacy and feasibility of an indicated depression-prevention intervention for non-professional caregivers with depressive symptoms administered through a smartphone app together with positive and corrective feedback through a telephone conference call.

## 2. Materials and Methods

### 2.1. Participants

A pretest–posttest design with a 1- and 3-month follow-up without a comparison group was used. Participants were recruited from the population of officially recognized (by the Administration) non-professional caregivers providing care for people in situations of dependency in the Autonomous Community of Galicia. To obtain a sample with similar characteristics to that of a future large-scale randomized controlled trial, the sample was selected by random stratified sampling following the following steps: (1) we contacted 11 regional and national associations related to caregivers via email and telephone, explained the purpose of the pilot study and obtained their cooperation; (2) every association made a list of all the non-professional caregivers they had, assigning a sequential number to each participant (e.g., 1, 2, 3,...); (3) a random number generator was used to select the sample, taking 10 participants per association (i.e., 110 random numbers were generated). The staff of every association contacted the randomly selected caregivers personally and put them in contact with the research team, who explained to them the purpose of the study and answered all their questions.

To participate in the study, they had to meet the following criteria: (a) be a non-professional caregiver for a family member in a dependent situation; (b) the dependent family member was officially recognized by the competent public bodies in Spain; (c) have a smartphone; (d) be at risk for depression, defined as a score equal to or greater than 16 on the Center for Epidemiologic Studies Depression Scale (CES-D; [38]; Spanish version [39]); (e) not meet the DSM-5 diagnostic criteria for a major depressive episode (American Psychiatric Association [40]); and (f) agree to participate in all evaluations. Exclusion criteria were: (a) having received psychological or psychopharmacological treatment in the last two months prior to study entry; (b) presenting with other disorders that could act as confounding variables (e.g., symptoms due to substance use); (c) presenting with serious mental health or medical disorders that require immediate intervention (e.g., suicidal ideation) or that make the study impossible (e.g., significant cognitive impairment, severe visual deficit); (d) the dependent family member had a very poor prognosis for the next 6 months; and (e) the caregiver anticipated a change of address or institutionalization of the dependent family member.

Of the 110 caregivers contacted, 101 agreed to take the eligibility assessment. Of these, a total of 33 (32.7%) met the eligibility criteria and were invited to participate in the study, and 2 (6.1%) of these refused to participate due to personal issues and difficulty handling the smartphone (93.9% participated in the pilot study). The final sample consisted of 31 caregivers (93.5% women with a mean age of 54.0 years); no dropout occurred. There were no differences between participants and those who refuse participation on sex (*p* = 1.000, Fisher exact test), age (U = 24.00; z = −0.529; *p* = 0.597), marital status (*p* = 0.216, Fisher–Freeman–Halton test), social class (*p* = 0.557, Fisher–Freeman–Halton test), monthly household income (*p* = 0.097, Fisher–Freeman–Halton test), level of education (*p* = 1.000, Fisher–Freeman–Halton test), primary activity (*p* = 0.432, Fisher–Freeman–Halton test), care recipient sex (*p* = 0.489, Fisher exact test), care recipient age (U = 16.50; z = −1.094; *p* = 0.274), relationship to caregiver (*p* = 0.424, Fisher–Freeman–Halton test), care recipient diagnosis (*p* = 0.318, Fisher–Freeman–Halton test), daily hours of care (U = 20.50; z = −0.814; *p* = 0.416), care duration (U = 11.50; z = −1.477; *p* = 0.140), depressive symptoms (U = 20.50; z = −0.796; *p* = 0.426), positive environmental reinforcement (U = 29.50; z = −0.114; *p* = 0.909), or negative automatic thoughts (U = 29.00; z = −0.151; *p* = 0.880).

Participation was voluntary and no financial or other incentives were provided. The confidentiality of the participants was guaranteed and all gave their informed consent. The investigation was conducted following the principles of the Declaration of Helsinki and was approved by the Bioethics Committee of the University of Santiago de Compostela (Code number 07/09/2016).

### 2.2. Instruments

The participants were evaluated at the pre- and post-intervention time points and the 1- and 3-month follow-ups. Data was collected through self-administered instruments through the smartphone app by the participants, and by a structured clinical interview administered via telephone by two independent evaluators. These interviewers were previously trained specifically for this study by two experts with more than 25 years of experience in evaluation and were unaware of the purpose of the study and the administered intervention.

This study used an ad hoc questionnaire to evaluate the sociodemographic variables and the care situation. The Structured Clinical Interview for DSM-5—Clinician Version (SCID-5-CV; [41]) was used to evaluate episodes of major depression, which offers good test-retest reliability for psychiatric patients (kappa index > 0.80 for all disorders). CES-D was administered to assess depressive symptoms ([38]; Spanish version [39]), with an internal consistency of 0.89. In order to assess the variables in the theoretical model in which the intervention was based, we used the Environmental Reward Observation Scale (EROS; [42]; Spanish version [43]), which evaluates positive reinforcement with an internal consistency of 0.86, and the Automatic Thoughts Questionnaire (ATQ; [44]; Spanish version [45]) which assess the frequency of negative automatic thoughts with an internal consistency of 0.96. The Client Satisfaction Questionnaire (CSQ-8; [46]; Spanish version [47]) was used to assess satisfaction with the intervention, with an internal consistency of 0.80.

Finally, to assess the acceptability of the interventions, data on attendance at sessions, and completion of assignments between sessions were collected through an ad hoc record worksheet prepared for this study.

### 2.3. Intervention

Before the study, a cognitive-behavioral intervention for indicated prevention of depression was adapted for application via a smartphone app. Previous studies had demonstrated that this intervention was effective in reducing the incidence of depression and depressive symptoms in the population of caregivers at short- and long-term when administered in a face-to-face group format [24,25] and in a conference call format [29]. This intervention is based on the Multifactorial Integrative Model of Depression developed by Lewinsohn et al. [48]. The adaptations consisted of modifying the content for presentation on smartphone screens (summarization and simplification of the content and changes to the format), designing the app interface, developing a daily reminder system that sent notifications to the mobile phone, and recording of between sessions homework tasks. The app-based intervention consisted of five modules that participants downloaded on their mobile and were programmed to be completed one per week. Module 1 explained the concept of depression and the need for active coping with depressive symptoms, and participants were trained in an activation control strategy (controlled breathing technique), self-reinforcement, and daily mood monitoring. Module 2 focused on how pleasant activities affect mood, and participants made plans to introduce them into their day-to-day activities. Module 3 addressed how thoughts affect mood, and participants were trained in techniques to manage negative thoughts. Module 4 explained how social contacts affect mood, and participants were trained in assertive communication and how to increase social contacts. In Module 5, participants reviewed everything they had learned and preventing relapse was addressed (see Table 1). The app, called Happy, was developed by independent programmers using the Apache Cordova framework, thus achieving a hybrid application, which is internally based on HTML, Javascript and CSS. For its development, an agile development methodology was followed. It was available for both Android and iOS in one language (Spanish) at the Google Play Store and the Apple App Store respectively, and operative for Android devices 4.4 or greater and for iOS 9 or greater. After the installation in the participant’s device, a user and password login previously facilitated by the research team was mandatory to access the app. The app offered participants the previously mentioned battery of questionnaires and, after being completed by the user, a notification with a summary of the results was sent. If the user met the eligibility criteria, they were invited to participate in the intervention by the research team. Every week, the app allowed access to the contents of the corresponding module, sending a message informing the participants that they could access the next module. Upon completion of the module, between-session tasks were assigned, and the app sent a daily notification to users reminding them to complete and record their intersessional homework tasks. The stored data was hosted on an internal server in a MySQL database protected under username/password, and which can only be accessed from an authorized host (only the research team, complying with all security and confidentiality guarantees).

In addition, a five-session intervention was manualized to establish telephone contact lasting approximately 30 min (1 session/week) with the participants in a group format (5–6 participants) using a conference call system. In these sessions, the group’s rules were explained and positive or corrective feedback was administered following the guidelines of Miltenberger (2012) [37]. The positive feedback consisted of providing information on how to correctly perform the homework tasks and providing reinforcement, and corrective feedback consisted of identifying the tasks that were not completed properly, suggesting relevant changes to improve performance.

The conference call intervention was administered by a psychologist and a doctor of psychology previously trained by experts with more than 25 years of experience in cognitive-behavioral therapies applied in different formats (e.g., individual, group) and through different means (e.g., face-to-face, telephone, online). The sessions were recorded, and adherence to the protocol was evaluated, yielding a protocol adherence of 92%. There were no significant differences between therapists in the results of the intervention.

### 2.4. Statistical Analysis

The quantitative data were analyzed by intention-to-treat. The missing values were imputed using the Last Observation Carried Forward Method. The McNemar test for paired data was used to analyze the incidence of depression and the risk of depression. Depressive symptoms were analyzed using repeated measures analysis of variance (ANOVA) and post hoc pairwise tests with Bonferroni correction. Student’s *t*-test for paired data was used to analyze the changes in depressive symptoms between the pre- and post-intervention time points and between pre-intervention and follow-ups, and changes in reinforcement and negative automatic thoughts at the pre- and post-intervention time points. The effect size was calculated using Cohen’s *d* (small = 0.2, medium = 0.5 and large = 0.8; [49]). Pearson’s correlation was used to analyze the association between positive environmental reinforcement, negative thoughts, and depressive symptoms at the post-intervention time point. Multiple linear regression analyses were performed to analyze variables that were predictive of a reduction in depressive symptoms, following the recommendations of Domenéch and Navarro (2006) [50]. Frequency analyses and descriptive statistics of sessions attended, tasks performed, and CSQ-8 scores were performed to analyze adherence and satisfaction with the intervention. The analyses were conducted using statistical package SPSS for Windows (version 22.0, IBMCorp., Armonk, NY, USA).

## 3. Results

### 3.1. Characteristics of the Sample

Table 2 presents the most relevant sociodemographic, care situation and clinical characteristics of the caregivers who participated in the study. The 93.5% of caregivers were women and the mean age of them was 54.0 years (range 41–71; Standard Deviation [*SD*] = 9.4). Of the caregivers, 93.5% lived with a partner; 58.1% declared that they belonged to a low/lower-middle social class; 58.1% had a monthly household income of between 1000 and 1999 Euros; 54.8% had a high school or university-level education; and 51.6% were not active members of the workforce (they were unemployed, did not work outside the home, or were retired).

In 51.6% of the cases, the family members receiving care were men, with a mean age of 54.0 years (*SD* = 28.1), ranging from 5 to 93 years. Most caregivers provided care to a family member, with the largest percentage being parents providing care for a child (38.7%). The most common type of illness observed among family members receiving care were mental health disorders, neurological diseases, and brain damage (48.4%). The time spent caring for the loved one was intensive and continuous. In this sample, caregivers devoted an average of 15.7 h a day to caring for their family member and had done so for an average of 15.9 years.

The mean depressive symptoms score was 26.8 (*SD* = 8.6). The mean positive environmental reinforcement score was 24.1 (*SD* = 4.1). The mean score for automatic negative thoughts was 70.7 (*SD* = 19.9).

### 3.2. Incidence of Depression

No participant developed an episode of major depression at the post-intervention time point or the one-month follow-up. Only two participants (6.5%) developed an episode of major depression at the three-month follow-up. Of them, one was diagnosed with an episode of major depression according to the DSM-5 diagnostic criteria, and one received an imputed value indicating depression.

Furthermore, there were no significant differences in the incidence of depression between the pre-intervention and post-intervention time points or between the pre-intervention time point and the one- and three-month follow-ups.

### 3.3. Depressive Symptomatology

**Figure 1:**
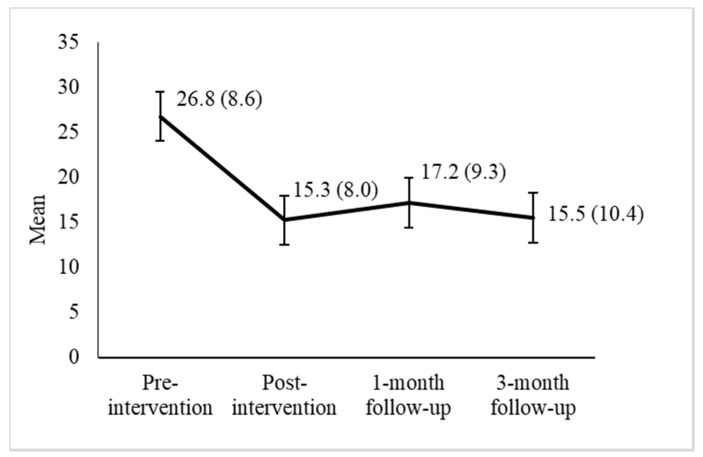
Depressive symptoms at the evaluation time points. The numbers indicate the mean Centre for Epidemiologic Studies Depression Scale (CES-D) score and the standard deviation is given in parentheses.

Figure 1 shows the progression of depressive symptom scores at the pre-intervention time point (Mean [*M*] = 26.8), post-intervention time point (*M* = 15.3), and one- and three-month follow-ups (*M* = 17.2 and *M* = 15.5, respectively). The repeated-measures ANOVA showed that changes in depressive symptom scores were statistically significant: *F*(3, 90) = 24.434, *p* < 0.001, *η*^2^ = 0.449. Compared to the pre-intervention time point, depressive symptoms were significantly lower at the post-intervention time point and at the one- and three-month follow-ups, with large effect sizes (Cohen’s *d* between 0.91 and 1.37; see Table 3).

In addition, 54.8% (*n* = 17) were not at risk of depression (i.e., CES-D score ≥ 16) at the post-intervention time point, 48.4% (*n* = 15) at the one-month follow-up, and 54.8% (*n* = 17) at the three-month follow-up. The decrease in the risk of depression between the pre-intervention and post-intervention time points was significant (*p* < 0.001), as was the difference between the pre-intervention time point and the one- and three-month follow-ups (*p* < 0.001 in both cases).

### 3.4. Variables for the Theoretical Model and Their Association with Depressive Symptoms

**Figure 2:**
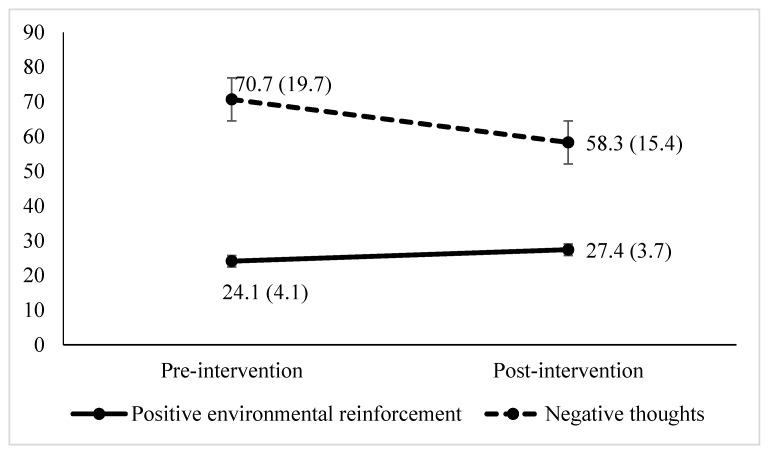
Positive environmental reinforcement and negative thoughts at the evaluation time points. The numbers indicate the mean scores and the standard deviations is given in parentheses.

Figure 2 shows the progression of positive environmental reinforcement and negative thoughts scores at the pre-intervention time point (*M* = 24.1 and *M* =70.7, respectively) and post-intervention time point (*M* = 27.4 and *M* = 58.3, respectively). After the intervention, the degree of positive environmental reinforcement from caregivers increased significantly: *t*(30) = −5.61, *p* < 0.001, with a large effect size (*d* = 1.01). The frequency of negative thoughts decreased significantly: *t*(30) = 3.47, *p* < 0.001, with a medium effect size (*d* = 0.62) (see Table 3).

Furthermore, a significant negative correlation was found between the degree of positive post-intervention environmental reinforcement and post-intervention depressive symptoms, *r*(31) = −0.61, *p* < 0.001. A positive correlation was also found between post-intervention negative automatic thoughts and post-intervention depressive symptomatology, *r*(31) = 0.59, *p* = 0.001.

### 3.5. Variables Predictive for the Results for Depressive Symptoms

The multiple linear regression analysis resulted in a significant model with an *R*^2^ = 0.295 (ETS = 7.07), *F*(3, 27) = 3760, *p* = 0.022. The level of pre-intervention depressive symptomatology was a predictor of post-intervention depressive symptomatology, *β* = 0.40, *p* = 0.033, 95% CI [0.33, 0.702].

### 3.6. Adherence and Satisfaction with the Intervention

Caregivers attended a mean of 4.8 sessions out of the five sessions that comprised the intervention (*SD* = 0.9). In this sample, 93.5% of caregivers attended all sessions. Furthermore, caregivers performed an average of 14.2 tasks out of the 18 assigned during the intervention and had a high degree of satisfaction with the intervention, with a mean score of 28.1 (*SD* = 3.9) on the CSQ-8.

## 4. Discussion

The objective of this pilot study was to evaluate the efficacy and feasibility of an indicated depression-prevention intervention administered through a smartphone app with the addition of conference-call contact aimed at non-professional caregivers with depressive symptoms. It was found that the incidence of depression, depressive symptoms, and the risk of depression decreased after the intervention. Furthermore, these changes were maintained at the three-month follow-up.

Only two caregivers (6.5%) had developed depression at the three-month follow-up. Although this incidence of depression is slightly higher than that found at the three-month follow-up in the previous study that used the same intervention in a face-to-face format [26] and conference call format [51], the numbers are nevertheless encouraging. In fact, they are less than 15.5% and 15.9% found in the control groups at the same follow-up time in the studies by Vázquez et al. [24,26].

There was also a significant reduction in depressive symptoms after the intervention, which continued until the three-month follow-up, with a large effect size (*d* = 1.37), which was larger than the effect size at the three-month follow-up following the face-to-face intervention format (*d* = 1.01) [26], but less than the effect size for the conference call format (*d* = 1.46) [51]. The mean score was reduced to 15.5 points, slightly below the threshold for risk of developing clinical depression, which is considered to be a score of 16 or more. Although this is consistent with other depression-prevention interventions [52], the mean score at the three-month follow-up was higher than that found using the same intervention delivered in face-to-face and conference call format [26,51]. On the other hand, it led to a significantly lower percentage of participants being at risk of developing depression, a figure that went from 100% at the pre-intervention time point to 45.2% at the post-intervention time point, though this is not as low as when the intervention is delivered face-to-face (29.5%) [25] and group conference call (18%) [51]. One possible explanation for these findings may be that the baseline level of depressive symptoms was greater in this study (*M* = 26.8) than for the studies examining the face-to-face format (*M* = 23.7) [26] and the conference-call telephone format (*M* = 22.4) [51]. In addition, administering the intervention through an app could also have some impact. While the face-to-face intervention or conference call-only format is more interactive, the smartphone app format of the intervention depends to a great extent on the caregiver’s self-determination and autonomy to read the texts with full attention and interpret them appropriately. Depending on their profile, certain caregivers may benefit more from the program if more human contact is added to the intervention program (such as reviewing the content read on the app during conference calls or increasing the duration of conference call sessions).

Regarding the variables in the theoretical model based on which the intervention was developed, a significant increase in positive environmental reinforcement was found after the intervention with a large effect size (*d* = 1.01), along with a significant decrease in negative thoughts with a medium effect size (*d* = 0.62). These results are consistent with the formulation of the multifactorial integrative model of depression developed by Lewinsohn et al. (1985) [48]. Furthermore, the correlations found between post-intervention positive environmental reinforcement, negative thoughts, and depressive symptoms suggest that there is a relationship between the variables in the theoretical model and depressive symptoms, consistent with the proposed model by Lewinsohn et al. (1985) [48]. However, these results should be taken with caution because the variable associations are not synonymous with causality. The changes in the variables from the theoretical model achieved with the intervention could have had an effect on depressive symptoms, but it is also possible that the change in depressive symptoms affected the variables from the theoretical model (positive environmental reinforcement and negative thoughts).

Those caregivers who had fewer depressive symptoms at the pre-intervention time point had better results after the intervention. One possible explanation is that the therapeutic change is more achievable when depressive symptoms are in their initial phase. Therefore, it is of utmost importance to administer preventive interventions in populations with the initial signs of depressive symptoms to prevent future depression.

Furthermore, adherence to the intervention was high. In this sample, 93.5% of caregivers attended all weekly conference call sessions. It is possible that telephone administration of the intervention, the short duration of the weekly telephone calls (only 30 min), the duration of the intervention (only five weeks), and adjusting to times that were convenient for them facilitated caregivers’ attendance at these sessions. Caregivers performed an average of 14.2 homework tasks out of the 18 assigned during the intervention. Notifications to record tasks sent from the app itself to the caregiver’s smartphone may have motivated and reminded the caregivers to complete and record the tasks. Lastly, satisfaction with the intervention was high, with a mean of 28.1 out of a maximum of 32 on the CSQ-8. Thus, participants rated the quality of the program very positively and indicated that it helped them better manage the difficulties that they experienced on a day-to-day basis.

This study has important implications for research and clinical practice. This is the first study on a psychological intervention for the indicated prevention of depression aimed at non-professional caregivers and administered through a smartphone app. Its innovative format and conference call delivery increases the accessibility of interventions and gives therapists new tools to reach more patients, following the recommendations of the National Institute of Mental Health Psychosocial Intervention Development Workgroup (2002) [53] and the New Freedom Commission on Mental Health (2003) [54]. It allows caregivers to receive a psychological intervention program at the most convenient time and place for them. Its duration is short (only five weeks), and the non-face-to-face and group format of the conference calls makes the intervention more cost-efficient, enabling reduced waiting lists and lowering the cost to the healthcare system. In addition, this intervention could prevent the personal suffering associated with depression. If its long-term efficacy is demonstrated in a randomized controlled trial as in the previous face-to-face interventions [26], an intervention of just five modules (via application) could prevent (or at least delay) the onset of depression for 12 months. Later, if depressive symptoms of the caregiver increase, we recommend the caregiver to review the contents learned or conduct reinforcement sessions to refresh the learned skills. If a caregiver develops a major depressive episode, they could resort to appropriate individual treatment in the healthcare system, allowing a clinical step-by-step care [32].

However, the present study has some limitations, mainly its lack of a control group and the corresponding randomization of the participants to the treatment conditions, as well as its small sample size. Likewise, it should be noted that a three-month follow-up could be too soon to draw definitive conclusions on the prevention of depression. Therefore, additional extended follow-ups are recommended. Lastly, although the smartphone app intervention format offers many advantages thanks to its accessibility, we do not know under what conditions the participants interact with the app. We did not have information about how much attention they paid to the texts, if there were distractions while interacting with the app, or how they interpreted the contents of the program. Certain caregivers, depending on their profile, may benefit more from a program with the addition of more human contact.

Despite the limitations, and consistent with the methodological recommendations made by Muñoz et al. (1996) [55] for studies on the prevention of depression, this study included aspects that made it a rigorous scientific study, such as the following: defining the population for which the intervention was designed (inclusion and exclusion criteria; defining the symptoms or objective condition); using specific, replicable, and manualized interventions; assessing therapists’ adherence to the protocol; using reliable and valid outcome measures; conducting multimodal evaluations; and blinding evaluation of results.

## 5. Conclusions

The results of the intervention considered here indicate a reduction in the incidence of depression and depressive symptoms, as well as a significant improvement of positive environmental reinforcement and negative automatic thoughts. Moreover, the high adherence and satisfaction with the intervention shows its acceptability and feasibility. The strengths of this intervention include its brief and innovative format via smartphone app and conference call, increasing its accessibility, flexibility and cost-efficiency, appealing to caregivers and therapists. This innovation could change the way in which psychological interventions are administered and reach a greater number of users, benefiting both caregivers from the present and the future. Taking into account the previously mentioned limitations (a lack of control group and a small sample size), these findings encourage future research of a randomized controlled trial with a larger sample size, and long-term follow-up to confirm the results of this study.

## Figures and Tables

**Table 1:** Contents of the brief intervention program.

Module	Content
Module 1	Presentation of group members
	Purpose of the program
	Information about depression and active coping of symptoms
	Mood rating
	Training in diaphragmatic breathing
	Self-reinforcement
	Homework: Mood rating, practice breathing control technique, self-reinforcement
Module 2	Explanation of the relationship between pleasant activities and mood
	Guidelines and strategies to increase pleasant activities
	Planning pleasant activities
	Behavioral contract
	Homework: Mood rating, practice breathing control technique, self-reinforcement, perform the planned pleasant activities
Module 3	Explanation of the relationship between thoughts and mood
	Thought management techniques (direct approach, priming, distraction)
	Planning pleasant activities
	Behavioral contract
	Homework: Mood rating, practice breathing control technique, self-reinforcement, perform the planned pleasant activities, practice the thought management techniques
Module 4	Explanation of the relationship between social contact and mood
	Guidelines and strategies to increase and improve social relationships
	Planning pleasant and social activities
	Homework: Mood rating, practice breathing control technique, self-reinforcement, perform the planned pleasant activities, practice the thought management techniques, make social contacts
Module 5	Review of what was learned. Maintaining progress
	Relapse prevention
	Farewell and closure

**Table 2:** Sociodemographic, care situation and clinical characteristics for the sample.

Sociodemographic Variables	*n* = 31	*%*
**Sex**		
Men	2	6.5
Women	29	93.5
**Age**		
*M*	54	
*SD*	9.4	
Range	41–71	
**Marital status**		
With partner	29	93.5
No partner (single, separated, divorced, widowed)	2	6.5
**Social class**		
Low/lower middle	18	58.1
Middle/upper middle	13	41.9
**Monthly household income**		
Up to €999	7	22.6
Between 1000 and 1999 euros	18	58.1
€2000 or more	6	19.3
**Level of education**		
Up to primary school education	14	45.2
Secondary/University	17	54.8
**Primary activity**		
Active worker	15	48.4
Unemployed/retired/housework	16	51.6
**Care situation variables**		
**Care recipient sex**		
Male	16	51.6
Female	15	48.4
**Care recipient age**		
*M*	54	
*SD*	28.1	
Range	5–93	
**Relationship to caregiver**		
Son/daughter	12	38.7
Father/mother	9	29
Other family members	10	32.3
**Care recipient diagnosis**		
Mental/neurological disorders/brain damage	15	48.4
Diseases of the skeletomuscular/connective tissue/cardiovascular/respiratory systems	7	22.6
Chromosomal/congenital/perinatal abnormalities	5	16.1
Others	4	12.9
**Daily hours of care**		
*M*	15.7	
*SD*	7.6	
**Care duration (years)**		
*M*	15.9	
*SD*	11.9	
**Clinical variables**		
**CES-D Score**		
*M*	26.8	
*SD*	8.6	
**EROS Score**		
*M*	24.1	
*SD*	4.1	
**ATQ Score**		
*M*	70.7	
*SD*	19.9	

M = Mean; SD = Standard Deviation; CES-D: Centre for Epidemiologic Studies Depression Scale; EROS: The Environmental Reward Observation Scale; ATQ: Automatic Thoughts Questionnaire.

**Table 3:** Student-*t* and Cohen’s *d* values between the pre-intervention and post-intervention scores, and at the one- and three-month follow-up measurements (*n* = 31).

	*t*	*p*	*d*	95% CI	
Variables				Minimum	Maximum
**Depressive symptomatology (CES-D)**					
Pre-intervention vs. post-intervention	7.62	<0.001	1.37	0.87	1.86
Pre-intervention vs. one-month follow-up	5.09	<0.001	0.91	0.49	1.33
Pre-intervention vs. three-month follow-up	6.89	<0.001	1.24	0.76	1.7
**Positive Environmental Reinforcement (EROS)**					
Pre-intervention vs. post-intervention	−5.61	<0.001	1.01	0.57	1.43
**Automatic negative thoughts (ATQ)**					
Pre-intervention vs. post-intervention	3.47	<0.001	0.62	0.23	1

95% CI: 95% Confidence Interval; CES-D: Centre for Epidemiologic Studies Depression Scale; EROS: The Environmental Reward Observation Scale; ATQ: Automatic Thoughts Questionnaire.
